# Editorial: Venoms, animal and microbial toxins, volume II

**DOI:** 10.3389/fphar.2022.973628

**Published:** 2022-08-19

**Authors:** Zhijian Cao, Delavar Shahbazzadeh, Hervé Kovacic, Patrick Michael McNutt, Jing-Lin Wang, Heike Wulff, Yuri Utkin, Jean-Marc Sabatier

**Affiliations:** ^1^ College of Life Sciences, Wuhan University, Wuhan, China; ^2^ Pasteur Institute of Iran (PII), Tehran, Iran; ^3^ UMR7051 Institut de NeuroPhysiopathologie, Aix-Marseille Université, Marseille, France; ^4^ Wake Forest Institute for Regenerative Medicine, Wake Forest School of Medicine, Winston-Salem, NC, United States; ^5^ Beijing Institute of Microbiology and Epidemiology, Beijing, China; ^6^ Department of Pharmacology, University of California, Davis, Davis, CA, United States; ^7^ Laboratory of Molecular Toxinology, Shemyakin-Ovchinnikov Institute of Bioorganic Chemistry RAS, Moscow, Russia

**Keywords:** venom, animal toxin, plant toxin, bacterial toxin, viral toxin, structure

Our Research Topic titled *Venoms, animal and microbial toxins, volume II* is centered on the characteristics of animal, plant, microbial toxins, and their molecular/cellular targets. It also addresses whole animal venoms, which contain a complex mixture of diverse toxins. Venomous animals and microbes are the natural sources of many toxins. These toxins vary in size, nature, and mode of action. They mainly act on ion channels, enzymes, receptors, and neurotransmitter release to produce an acute pathophysiological effect. Animal venom and microbial molecules behave as candidate therapeutics or biological weapons because of their unique potencies, rapid mode of action and wide range of bioactivities.

This Research Topic compiles twenty research and review articles to describe venoms and toxins (or derivatives), through an “in-depth” analysis of their structure, pharmacology, synergistic effects, and structure-function relationship.

Several articles are focused on the structural and/or functional characterization of animal and microbial toxins, as well as other animal venom compounds. A first article by Ullah et al. describes the three-dimensional (3-D) structure of the exfoliative toxin D (ETD) from the pathogenic bacterium *Staphylococcus aureus* responsible for skin disorders. The authors predicted the 3-D structure of ETD using optimized molecular modeling techniques, and compared it to those of four other known exfoliative toxins (A, B, C and E). The authors then used the predicted ETD structure for *in silico* docking simulations of natural and synthetic inhibitors, which is important to inform the design of new potent inhibitors to treat staphylococcal scalded skin syndrome. The article by Peng et al. reports on the isolation and characterization of δ-theraphotoxin-Gr4b, a novel toxin (37-residue peptide crosslinked by 3 disulfide bridges) from the venom of the spider *Grammostola rosea*. Interestingly, this toxin inhibits the fast inactivation of the Na_v_1.9 channel through a unique mechanism: binding to domains III and IV voltage sensors. Since Na_v_1.9 plays a key role in pain perception, these finding may inform a therapeutic development of novel analgesics. In another article, Dongol et al. screened venoms from Australian theraphosid spider species against the human pain target hNa_V_1.7 and isolated Ssp1a, a 33-residue peptide with an “inhibitor cystine knot” motif. Functional characterization of recombinant Ssp1a on neuronal hNav subtypes suggested Ssp1a interacts with the voltage-sensing domain II of hNa_V_1.7 to trap the channel in a resting state. George et al. investigated the effects of synthetic peptide toxins predicted from Arizona bark scorpion venom on the voltage-gated sodium channel Na_v_1.8, another channel involved in pain signaling. The authors found the peptide NaTx36 inhibited sodium current recorded from a recombinant grasshopper mouse Na_V_1.8 channel (OtNa_V_1.8) via interactions with the domain I voltage sensor and domain II pore module. Ho et al. described the structural and functional properties of an α-conotoxin OmIA isolated from *Conus omaria* venom. OmIA behaves as a potent antagonist of α7 nicotinic acetylcholine receptors (nAChRs). The authors analyzed a co-crystal structure of OmIA with the acetylcholine binding protein of gasteropod *Lymnae stagnalis*, and highlighted key amino acid residues responsible for the high potency of OmIA against these receptors. Huynh et al. characterized α-elapitoxin-Oh3a, a 72-residue post-synaptic neurotoxin derived from venom of the Asian *Ophiophagus hannah* king cobra. This toxin, which causes neuromuscular paralysis, inhibited the contractile responses of tissues to exogenous carbachol and acetylcholine in chick *Biventer cervicis* nerve-muscle samples. King cobra antivenom prevented α-elapitoxin-Oh3a-induced neurotoxicity *in vitro*, suggesting an active role for α-elapitoxin-Oh3a during king cobra intoxication. Yu et al. focused on *Nemopilema nomurai* jellyfish envenomation, a threat to humans in Asian waterways. Metalloproteinases are reportedly the main toxic components of the venom, causing inflammation and damage. The authors isolated and characterized high proteinase activity fractions in tentacle autolysis using complementary purification techniques. Unexpectedly, these fractions did not show any proteinase activity except when mixed together, highlighting some synergistic effects among components of the fractions. Elrayess et al. characterized Smp24 and Smp43, two “novel” cationic antimicrobial peptides (AMPs) from the venom of the scorpion *Scorpio maurus palmatus*. The authors examined peptide-induced cytotoxicity on various cell lines, including leukaemia and non-cancer cells. The peptides were found to alter the viability of all cells tested (although HaCaT human skin keratinocytes were less sensitive). This decreased cell viability was accompanied by a selective up-regulation of the caspase-1 gene (except for HaCaT cells), whereas all tested cells showed an increase in downstream interleukin-1β expression. These data suggested scorpion venom AMPs activate pyroptosis, a highly inflammatory signaling cascade leading to a lytic programmed cell death.

Other articles of the Research Topic are focused on the structural characteristics, mode of action, targets and fields of application of toxins, venom components or whole venoms. For example, Wu et al. characterized FM-CATH, a “novel” cathelicidin from the *Fejervarya multistriata* paddy frog skin. FM-CATH has potent antimicrobial properties against both bacteria and fungi. It binds to lipopolysaccharides and lipoteichoid acid, and induces agglutination of bacteria. It also alters enzymatic activities (plasmin, thrombin, tissue plasminogen activator, β-tryptase) thus inhibiting the coagulation process both *in vitro* and *in vivo*. FM-CATH increased survival of septic mice, suggesting it might be of value in the treatment of sepsis. Wang et al. studied PcActx, a toxic peptide from the zoantharian *Palythoa caribaeorum* with potential inhibitory activity on the transient receptor potential cation channel subfamily V member 1 (TRPV1). TRPV1 conducts Ca^2+^, is widely expressed in sensory neurons but is also expressed in epileptic brain areas and is thought to be a potential target to prevent epileptic seizures. At non-toxic doses, PcActx peptides (reduced and folded oxidized forms) reversed pentylenetetrazol-induced seizure-related behavior in zebrafish larvae by limiting the production of reactive oxygen species (ROS) and modulating the expression of genes involved in Ca2+ and GABA-glutamate signaling. The authors conclude PcActx is a potential novel treatment for epilepsy. Another study by Wu et al. focused on Cath-MH, an AMP from the skin of the frog *Microhyla heymonsivogt*. The authors investigated the antimicrobial potential of Cath-MH on *Propionibacterium acnes*. Cath-MH had bacteriocidal effects through membrane disruption. *In vivo*, Cat-MH showed agglutination activity, and reduced edema and infiltration of inflammatory cells in an acne mouse model. Cath-MH also suppressed bacterial growth and production of pro-inflammatory cytokines *in vivo*, suggesting it might be useful to manage acne vulgaris and related skin disorders. Gutierres et al. examined the protective effects of the phospholipase A2 inhibitor Varespladib on the deleterious actions of the venom from the southern American bushmaster *Lachesis muta rhombeata* pit viper snake. The enzymatic, coagulant and hemorrhagic activities of venom were studied in the presence of varespladib (with or without addition of a commercial antivenom). Varespladib potently antagonized PLA2 activity preventing venom-induced coagulation, whereas it had little or no effects on esterasic, caseinolytic, and hemorrhagic activities. Eisele et al. studied the binary C2 toxin of the highly pathogenic *Clostridium botulinum* bacteria. The binary C2 toxin consists of two proteins: C2I (enzyme) and C2II (binding/transport) subunits. To exert toxic effects on mammal cells, C2II needs to be proteolytically cleaved to the pore-forming subunit C2IIa. The authors demonstrate that C2IIa reduces the chemotactic translocation of human neutrophils (polymorphonuclear leukocytes), thus have potential to down-modulate the excessive and deleterious recruitment of neutrophils into organs after trauma. Barros et al. reviews bioactive peptides and alkaloids identified in skin secretions of Urodele amphibians, which include antimicrobials, antioxidants, immune system modulators, vasoactive and coagulation-acting compounds and which could serve as “new” scaffolds for drug design.

Another set of articles of the Research Topic are focused on the potential treatment of envenomation using antibodies or other compounds. Johnston et al. introduced the “Australian Snakebite Project” (ASP-24), and detailed the epidemiology and clinical presentation of Australian sea snake envenoming. The efficacies of antivenoms in preventing myotoxicity and neurotoxicity are presented. The morbidity and mortality related to sea snake envenoming are discussed and it is concluded that early antivenom treatment after host envenomation is a key therapeutic intervention to prevent severe myotoxicity and death. Hmaidi et al. described the molecular basis of the interaction between the cardiotoxic α-neurotoxin AahII (scorpion *Androctonus australis hector*) and the (cardiac) voltage-gated Na_v_1.5 channel. The authors showed that AahII slows the fast inactivation of Na_v_1.5 channels expressed in HEK293 cells. A highly neutralizing anti-AahII nanobody (previously produced Nb10) was shown to fully reverse the effects of AahII on the kinetics of channel inactivation. Computer-aided docking experiments suggest that at AahII molecule, Nb10 might bind to the same binding sites as Na_v_1.5. Sachetto et al. studied the neutralization of toxins in venom from the lethal Brazilian pit viper *Bothrops jararaca*. The bioflavonoids rutin and its water-soluble derivative rutin succinate were used to assess their protective potential against the snake venom in *in vitro* and *in vivo* assays (*in vivo*, mice were injected with venom, or venom preincubated with rutin or rutin succinate). The data indicated that both flavonoids prevent venom-induced lethality through multiple mechanisms (e.g., coagulation, metalloproteinases). Interestingly, rutin, and rutin succinate showed different modes of action on homeostasis, which would deserve a more detailed analysis of the structure-activity relationships. A similar approach was followed by Heber et al. using ambroxol to neutralize exotoxins TcdA and TcdB from the enterobacterium *Clostridioides difficile*. TcdA and TcdB are the main virulence factors produced by the bacterium and the cause of *C. difficile* associated diseases (CDAD). To exert their toxic effects, the two exotoxins are internalized into the cells via receptor-mediated endocytosis. Translocation of exotoxins from endosomal vesicles into the cytosol requires the acidification of endosomes; ambroxol prevents such acidification. The authors therefore examined the potential protective effects of ambroxol on TcdA- and TcdB-induced cytotoxicity. Ambroxol was found to inhibit key (exotoxin-induced) events (i.e., endosome acidification, morphological changes, glucosylation of Rac1), whereas it also unexpectedly decreases the intracellular enzyme activity of exotoxins. Ambroxol thus behaves as a candidate therapeutic against CDAD. Silva et al. discussed translational concerns regarding the use of rodent lethality models to evaluate antivenoms for human envenoming. To illustrate this problem, it was shown that human nicotinic acetylcholine receptors (nAChRs) have an exceptionally low affinity for the short-chain α-neurotoxins compared to long-chain α-neurotoxins, while both types of α-neurotoxins bind to mouse nAChRs with high affinity. The authors pointed out that the effects of purified toxins or animal venoms on natural prey species are likely to be different from the effects on non-prey species, including humans.

A final article by Hirschenberger et al. describes CRISPA, a transient and non-viral technique to deliver Cas9 endonuclease into specific cells. The strategy developed by the authors is based on the translocation machinery of the *Bacillus anthracis* anthrax toxin, PA (protective antigen). The PA transporter, which normally mediates the entry of anthrax lethal factor and edema factor into the cells, might be optimized for a cell-type specific delivery of Cas9. Therefore, CRISPA potentially represents a step forward, in the translation of the CRISP/Cas9 genome editing technology into clinics.

Taken together, Volume II of this Research Topic contributes to a better understanding of venoms, venom compounds and toxins (or derivatives) opening the way to new exciting research and discoveries. We do believe that these articles exploring a particularly complex world, will inspire researchers and clinicians worldwide.

